# A Mechanistic Study on the Fluidization and Enhancement Effects of Cineole Toward Stratum Corneum Intercellular Lamellar Lipids: A Liquid Crystalline Model Approach

**DOI:** 10.5812/ijpr-134731

**Published:** 2023-06-22

**Authors:** Leila Karami, Hamid Reza Moghimi

**Affiliations:** 1 Department of Pharmaceutics and Pharmaceutical Nanotechnology, School of Pharmacy, Shahid Beheshti University of Medical Sciences, Tehran, Iran

**Keywords:** Lamellar Liquid Crystals, Skin Permeation Enhancers, 1,8-Cineole, Transdermal Drug Delivery, Model Stratum Corneum Lipids

## Abstract

**Background:**

The stratum corneum (SC) serves as the primary barrier for permeation in human skin. Penetration enhancers, such as 1,8-cineole, are utilized to enhance the permeation of drugs. Cineole increases the permeation of chemicals through different mechanisms. However, its mechanism, particularly at high concentrations, has not been well-studied and is the subject of the present investigation.

**Objectives:**

In continuation of our previous studies, the present investigation aims to elucidate the mechanism of action and concentration dependency of the effects of 1,8-cineole on the structure, diffusional properties, and partitioning behavior of the SC at high concentrations. This will be achieved through lamellar liquid crystalline models and ex-vivo skin studies.

**Methods:**

A lamellar liquid crystalline lipid matrix model in the presence (25 - 90%, w/w) and absence of cineole was prepared from SC lipids and characterized by X-ray diffraction, differential scanning calorimetry (DSC), thermogravimetric analysis (TGA), and polarized light microscopy (PLM) studies. Release of the model lipophilic drug (diazepam) from cineole and cineole-treated matrices and the permeation of the drug from cineole and cineole-containing matrices (as a vehicle similar to the stratum corneum lipids) through excised rat skin were studied. Drug assay was performed by HPLC.

**Results:**

The PLM, DSC, and X-ray studies showed that the model matrix had a lamellar gel-liquid crystalline structure, and cineole fluidized the structure concentration-dependently and created other mesomorphic textures, such as myelinic figures. Release experiments showed that diffusion coefficients remained almost constant at high cineole concentrations of 40-90%, suggesting similar fluidization states. Skin permeation studies indicated that the diffusion coefficient (estimated from lag-time) increased concentration-dependently and played a role in permeability coefficient (Kp) increments alongside the increased partitioning of the model drug into the skin. Data suggest that high concentrations of cineole at the skin surface might not provide enough cineole in the skin for full fluidization, despite the similarity of the vehicle to SC lipids and even at high concentrations.

**Conclusions:**

The enhancement effect of cineole is concentration-dependent and might reach maximum fluidization at certain concentrations, but this maximum might not be easily achievable when cineole is used in formulations as pure or in a vehicle.

## 1. Background

The outermost layer of the epidermis, the stratum corneum (SC), is the main permeation barrier of the skin (1). The SC is composed of keratin-rich flattened cells (corneocytes) embedded in an intercellular lipid matrix (2, 3), creating a unique wall-like structure (4) in which the intercellular domain seems to be the major rate-limiting pathway for the permeation of most drugs (5, 6). The excellent barrier properties of the SC often prevent transdermal drug delivery (7, 8). One approach to increase skin permeation is to use chemical penetration enhancers (7, 9), including terpenes (10-12), such as 1,8-cineole, the subject of the present investigation.

Many researchers have used cineole as a penetration enhancer for different biological barriers. A 3.5-fold increase in low molecular weight heparin absorption through human skin was observed by 10% cineole (13). Saffari et al. showed that cineole could increase cellular uptake and the biological effect of liposomal gene delivery in cancer (14). Moghimi et al. showed that the permeation of drugs through human burn eschar could be enhanced significantly by 1,8-cineole (15). Regarding the enhancement mechanism, Yamane et al. (16) and Williams and Barry (12) showed that cineole increased the permeation of 5-fluorouracil by about 95-fold, probably by disrupting the stratum corneum structure. In a series of works using a lamellar liquid crystalline structure as a model for human stratum corneum, Moghimi (17, 18) showed that cineole increased skin permeation of the model hydrophilic drug (fluorouracil) and model lipophilic drug (estradiol) by fluidization of intercellular lipids and changed their partition coefficients.

The same group employed a solvent model to show that cineole increased the partitioning of hydrophilic drugs in the hydrocarbon domain of intercellular lipids (19). Another mechanism is the creation of polar pathways by cineole in the intercellular lipids of the stratum corneum in skin permeation enhancement of hydrophilic drugs (20). The dominance of the above-mentioned mechanisms depends on the lipophilicity, concentration, and structure of both the terpene and the drug, and often a combination mechanism works (15).

Moghimi et al. (21) prepared a lamellar liquid crystalline SC model. In a series of investigations (17, 22), the influence of 5 - 25% cineole on the barrier properties and 5 - 40% cineole on the structure of the model was studied and compared to human skin data, indicating that the model can predict the effects of cineole on barrier properties of human skin. The outstanding point was that the structure and enhancement effects were strongly concentration-dependent (17, 22), and there was a balance between changes in diffusion coefficient and partition coefficient over cineole concentrations of 5 - 25% for both hydrophilic and lipophilic drugs (17, 18). Our previous results (22) also showed the presence of spherulites and myelinic figures in cineole-treated matrices at 25% and 40% cineole. Such structures are expected to affect the permeation and partitioning of drugs, increasing at higher cineole concentrations. Also, our previous observations showed a high degree of fluidization at 40% cineole. 

## 2. Objectives

The present study aimed to investigate the influence of higher concentrations of 1,8-cineole (40 - 90%) on diffusional and partitioning properties of the barrier by a mechanistic approach using model lipid matrix and ex-vivo skin studies for a lipophilic model drug.

## 3. Methods

### 3.1. Materials

Stearic acid (97%), myristic acid (99%), oleic acid (extra pure), and palmitoleic acid (> 98%) were purchased from Exir (Austria). Palmitic acid (98%) and linoleic acid (99%) were purchased from Acros Organic (USA). Cholesterol (> 99%) and butylhydroxytoluene (98%) were purchased from Sigma (Germany). Also, 1,8-cineole (99%) was supplied by Alfa Aesar (Germany). Diazepam was supplied by Changzhou Siyao Pharmaceutical Company (Changzhou, China). All other solvents and reagents were of analytical or HPLC grade.

### 3.2. Preparation of Lamellar Matrix

The model matrix containing fatty acids and cholesterol was prepared according to Moghimi et al. (21). In brief, fatty acids, cholesterol, and antioxidant were dissolved in a mixture of chloroform: methanol (2:1, v/v). The amount of fatty acids as a whole is 55% (w/w) of the model matrix, and those of cholesterol, water, and antioxidant are 20, 25, and 0.02 (%w/w) of the model matrix. The solvent was then evaporated by a rotary evaporator (Heidolph, Germany). An alkaline aqueous solution was then added to the lipid mixture to provide a water content of 25% (w/w) and partially neutralize about 40% of fatty acids. The flask was then sealed and mixed as described before (21).

Cineole-treated matrices (25, 40, 50, 60, 70, 80, and 90% w/w) and drug–containing matrices (40, 60, and 90% w/w cineole) were prepared by adding pure cineole and/or cineole solution of diazepam (model lipophilic drug) to the prepared matrix.

### 3.3. Thermogravimetric Analysis

Thermogravimetric Analysis (TGA) was performed to assess solvent evaporation efficacy and the water content of the matrix using TGA-50 (Shimadzu, Japan) over a temperature range of 25°C to 250°C at a heating rate of 5°C min^-1^ (21).

### 3.4. Karl Fischer Titration

To determine the water content of the model matrix, Karl Fischer titration (KFT) experiments were conducted on a KF Titrator (Mettler Toledo DL77, Switzerland) using the protocol described by Moghimi et al. (21) for the complete dissolution of the matrix samples.

### 3.5. X-ray Diffraction Studies

X-ray diffraction is appropriate for studying the structure of liquid crystals (23-25). Here, X-ray diffraction experiments were performed on matrices using a PANalytical diffractometer (X'Pert PRO MPD, PANalytical BV, the Netherlands) with a KαCu radiation and wavelengths of Kα_1_ and Kα_2_ of 1.54 Å at ambient temperature. Samples were put on zero-background silicon, and experiments were done over 2θ range of 3 - 80 (θ: scattering angle) with a step size of 0.0260° 2θ and a scan step time of 37.9950 s. Peak positions and corresponding repeat distances were obtained using PANalytical X'pert HighScore software version 3.0.5. (Malvern Panalytical, UK).

### 3.6. Polarized Light Microscopy

Mesomorphic textures and structures of systems were studied by cross-polarized light microscopy (PLM) using a Ceti Magnum-POL Polarization Microscope (Medline Scientific, UK) connected to a microscope camera (DFK MKU130, The Imaging Source, Germany) at room temperature.

### 3.7. Differential Scanning Calorimetry

Differential scanning calorimetry (DSC) studies were carried out employing a DSC-60 instrument (Shimadzu, Japan) using pressure-proof pans over a temperature range of -30 to 120°C and a heating rate of 5°C min^-1^ (21).

### 3.8. Release Studies

Release of diazepam from matrices or cineole was studied employing cellulose acetate membranes and home-made diffusion cells using 5% (v/v) hydroalcoholic solution (thermostated at 37°C) as the receptor phase. Samples of the receiving solutions were withdrawn at predetermined time intervals of up to 32 hours and replaced with an equal volume of receptor phase. Diazepam contents in the receiving samples were determined by HPLC, as described later. Diffusion coefficients of diazepam in the systems were calculated using Higuchi's equation from release data.

### 3.9. Skin Permeation Studies

Skin permeation studies used Franz-type diffusion cells and full-thickness Sprague-Dawley rat's abdominal skin. All skin experiments were performed according to the Ethics Committee of the Shahid Beheshti University of Medical Sciences with their permission. Through a painless technique for humane euthanasia of laboratory animals, rats were anesthetized, the hair was shortened using an animal grooming machine, sacrificed, and full-thickness skin was separated. Skin samples were washed thoroughly with deionized water, and underlying fats were removed, wrapped in aluminum foil sheets, and stored at -20°C until use.

For permeation studies, skin samples were thawed at room temperature, mounted on diffusion cells with the dermis side facing the receptor compartment and 5% (v/v) hydroalcoholic solution, thermostated, and stirred at 37°C to keep the skin surface temperature at 32°C. Diazepam-containing matrices or cineole solutions of diazepam were then mounted on the skin surfaces in the donor compartments. The matrices were used here as a vehicle similar to the stratum corneum lipids to facilitate the partitioning of cargo into the skin. Sampling and drug determination were as described above.

The skin data were analyzed according to Fick's Law. Hence, the slope of the linear part of the permeation graph (cumulative amount of drug permeated vs. time) was considered the flux (J), and the permeability coefficient (Kp) was calculated using the donor concentration (C) and Kp = J/C equation. According to Fick's Law and considering constant barrier thickness (h), Kp ratios would be the multiplication of diffusion coefficient ratios (D ratios) by partition coefficient ratios (Km ratios). As the diffusion coefficient is correlated to lag-time (L) through L = h^2^/(6D) equation, considering constant membrane thickness, D ratios would be reciprocal of lag-time ratios. Lag time was obtained as a time intercept of the permeation graph. As explained before, the diffusion coefficient in the model matrix was also calculated from release data.

### 3.10. Diazepam Assay

A reverse-phase high-performance liquid chromatography (RP-HPLC) system with a Merck-Hitachi separation module (Hitachi, Japan) coupled with a UV-visible detector (L-7420) was used for the quantitative assay of diazepam. Isocratic elution was performed on C18-MZ-Analitical Perfectsil Target, 250 × 4.6 mm column (MZ-Analytical, Germany) at a 1.0 mL/min flow rate and column temperature of 25°C. The mobile phase was a combination of water and acetonitrile (50:50). The run time was 16 min, and the retention time of diazepam was around 14 min. Drug standards were prepared in the mobile phase and detected at a wavelength of 254 nm (26).

### 3.11. Statistical Analysis

Data were analyzed using IBM SPSS Statistics version 26.0 software (IBM, Armonk, NY, USA). One-way Analysis of Variance (ANOVA) was used to determine whether the values differed statistically. The differences were considered significant at P ≤ 0.05.

## 4. Results

### 4.1. Water Content of Model Matrix and Solvent Evaporation Efficacy

**Figure 1.**
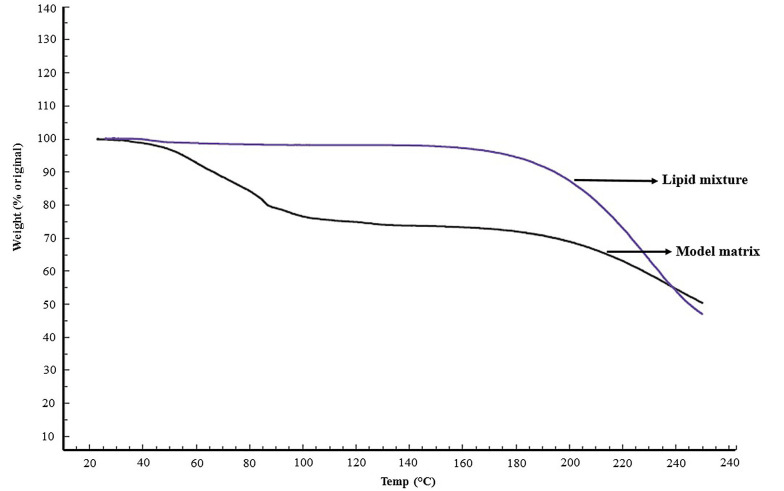
Thermogravimetric analysis of the lipid mixture prior to hydration and the model matrix, indicating efficient solvent evaporation from the lipid mixture and about 25% water content of the model matrix.

Thermogravimetric analysis of the lipid mixture prior to hydration (Figure 1) demonstrated a negligible weight loss of less than 1%, indicating efficient solvent evaporation during the preparation procedure. As expected, the model matrix demonstrated about 24.50 ± 2.2% (mean ± SD, n = 4) weight loss (Figure 1). Water assay of the model matrix by KFT revealed 24.15 ± 0.7% (w/w) (mean ± SD, n = 5) water content, in good agreement with TGA data and Moghimi et al. (21).

### 4.2. X-ray Diffraction Studies

**Figure 2.**
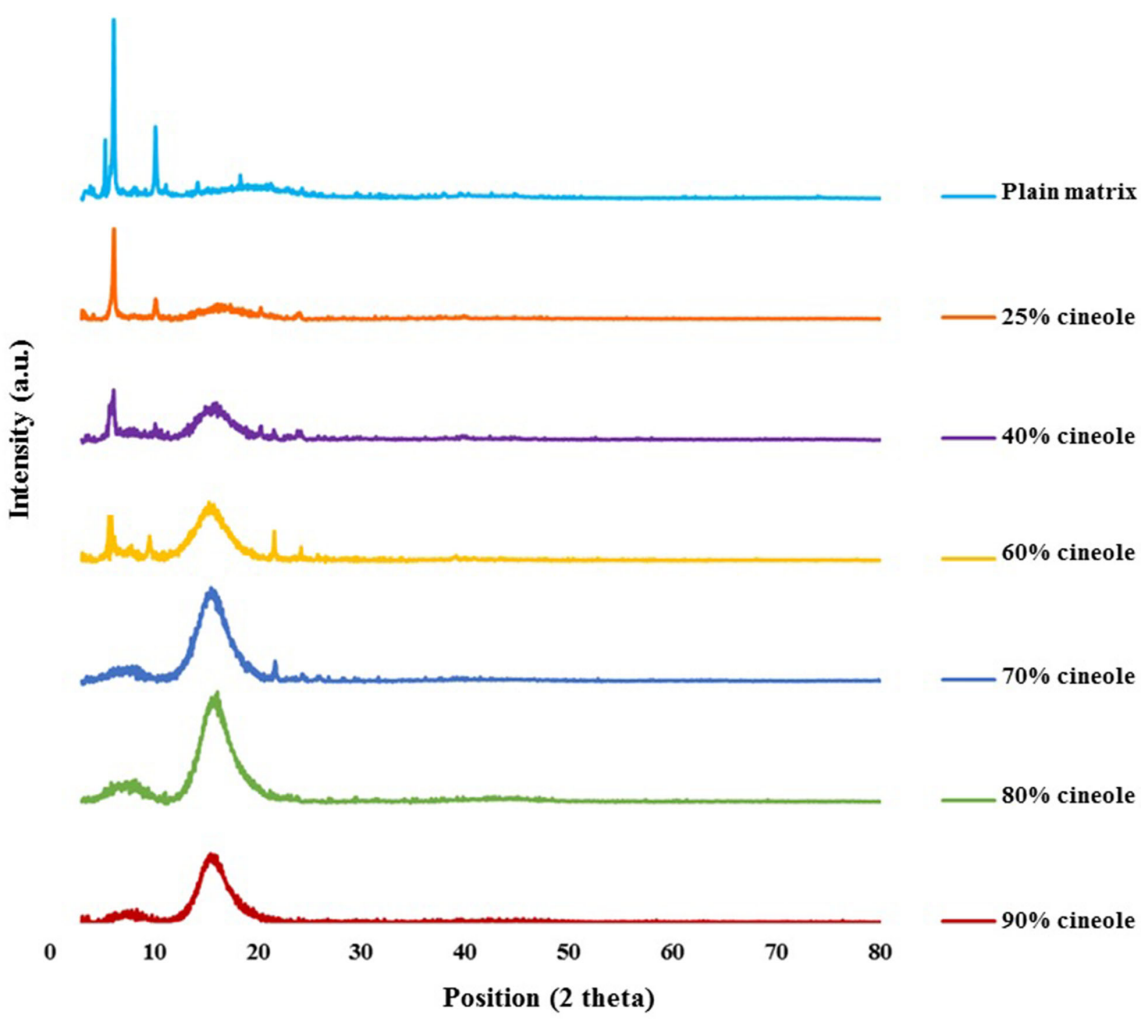
X-ray diffraction profiles of the plain (untreated) model matrix and cineole-treated matrices at different cineole contents (% w/w) at ambient temperature.

Figure 2 shows the reflection intensity versus 2θ profiles of the untreated model matrix and cineole-treated matrices at ambient temperature. Using the Bragg equation 2d sin θ =nλ, the repeat distances (d) were calculated from peak positions (2θ). The matrix shows multiple sharp reflections in the small-angle region (2θ < 10°), including those corresponding to d-spacings of 41.46, 32.29, 23.89, 21.24, 16.62, and 14.39 Å, in good agreement with Moghimi et al. (21). In the high-angle region, the matrix shows a broad continuous band around 2θ = 20°. In this region, sharp reflections at 2θs equal to 10.16, 21.2°, 22.8°, and 24.2° corresponding to d-spacings of 8.7, 4.2, 3.9, and 3.67 Å are also observed.

By adding 25 - 90% cineole to the model matrix, some changes were observed in the reflection profile of the model matrix (Figure 2). In samples containing 25% cineole, the intensity of reflections in the small-angle region was reduced, and the diffuse band in the high-angle region became more visible. In matrices containing 40% cineole, these changes were more apparent, and in matrices containing 70% and more cineole, the intensity of the sharp reflections was reduced intensely, and the diffuse band in the high-angle region became greater.

### 4.3. Polarized Light Microscopy

**Figure 3.**
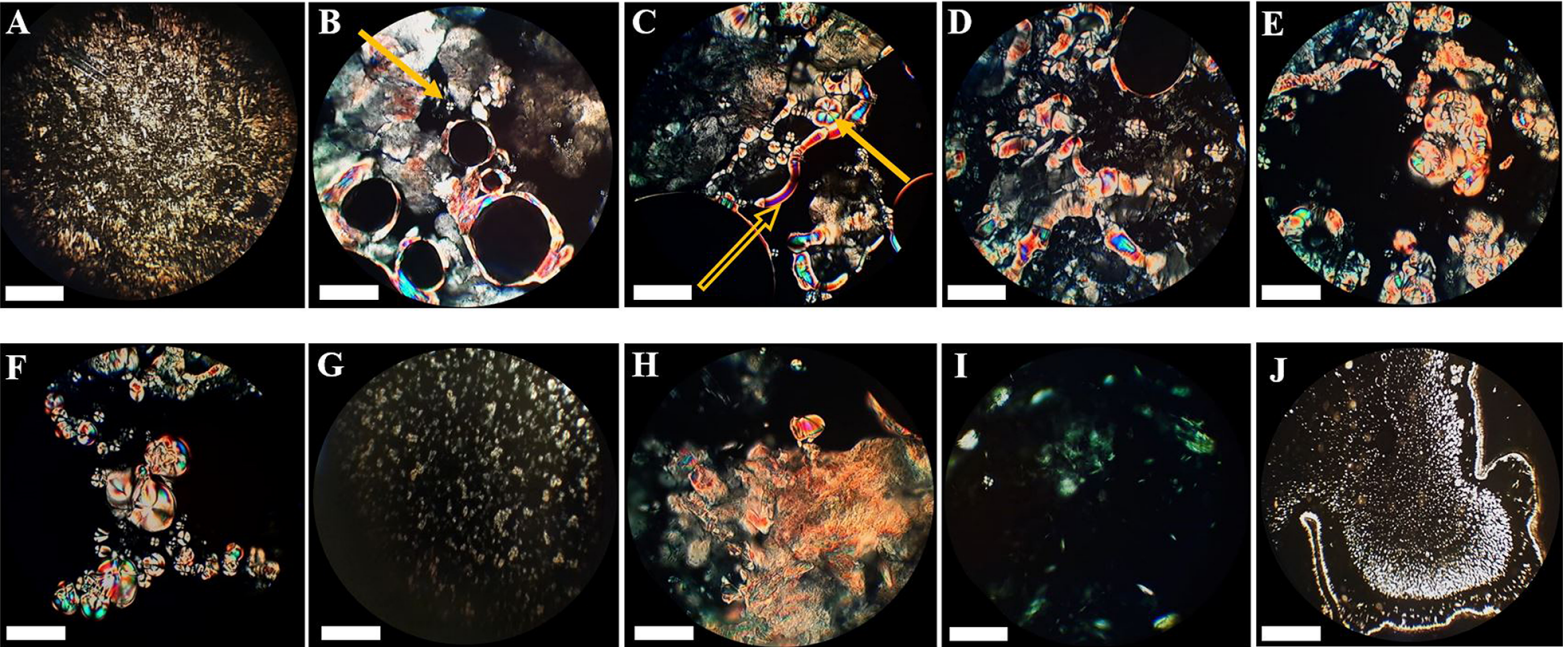
Photographs illustrating the textures of the model matrix and cineole-treated matrices at different cineole contents (% w/w) at ambient temperature. (A) Plain (untreated) matrix, (B) 25%, (C) 40%, (D) 50%, (E) 60%, (F) 70%, (G) liquid part of 80%, (H) and (I) bulky parts of 90% and (J) liquid part of 90%. A positive sample unit is illustrated in Figure B (pointed by an arrow), a myelinic figure in Figure C (pointed by a hollow arrow), and a spherulite in Figure C (pointed by a solid arrow). See text for more details. The scale bar represents 50 µm.

The model matrix showed oily streaks and mosaic textures in polarized light microscopy. Matrices containing 25% cineole showed structural changes, so the mosaic texture and positive units started growing. Increasing cineole content to 40% created more mosaic textures and positive units, replacing the oily streaks (Figure 3). Moreover, at this cineole concentration, spherulites were observed, as reported by Moghimi et al. (22). In systems containing 50% and 60% cineole, many positive units and myelinic figures showed up, and spherulites continued to develop. At 70% cineole, there were still positive units, myelinic figures, and spherulites (Figure 3). By increasing cineole to 80% and 90%, the matrix became two-phasic, an oily liquid phase and a bulky mass. Positive units were observed in the liquid phase, while in the bulky mass, there were many oily streak textures (Figure 3) with no myelinic figures and positive units.

### 4.4. Differential Scanning Calorimetry

**Figure 4.**
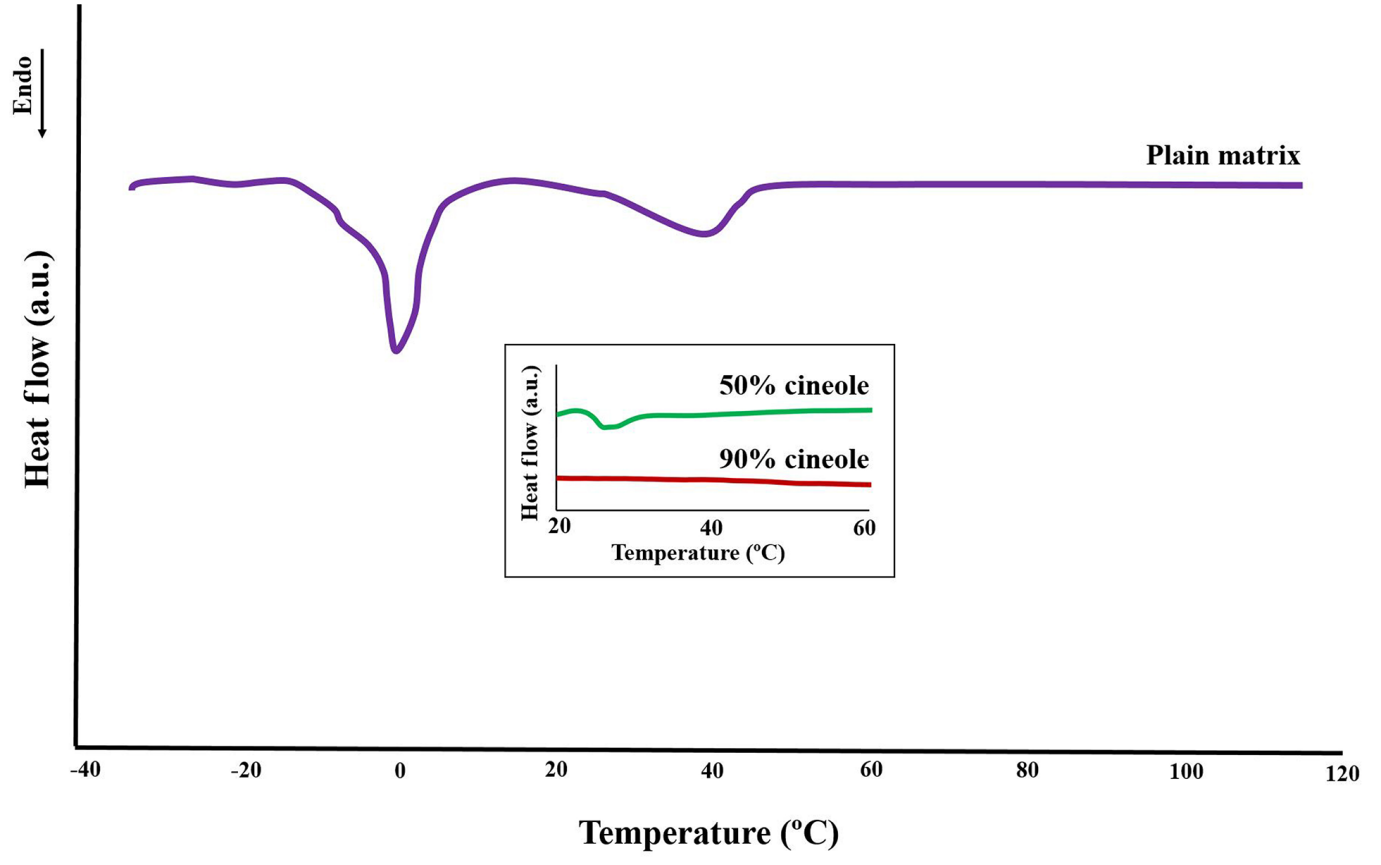
Differential scanning calorimetry profile of the plain model matrix showing several endothermic transitions from -30°C to 120°C. The effects of cineole on the main structural transition temperature at 40°C (related to the bilayer arrangements) are shown in the insert box, showing diminishes of this transition by increasing the cineole content of the model matrix. See text for details.

The thermogram of the plain model matrix (Figure 4) showed two main endothermic transitions at about 0°C and 40°C, as also reported by Moghimi et al. (21), of which the last one is related to the lamellar structure. Two other transition temperatures were reported by Moghimi et al. (21) at around -10°C and 21°C, both of which are seen here as shoulders of the 0°C and 40°C transitions (Figure 4). The addition of cineole at high concentrations (50% and 90%) caused a reduction in the intensity of the main structural transition of 40°C (insert box of Figure 4).

### 4.5. Release Studies

Diffusion coefficients of diazepam in matrices containing 40 - 90% cineole and pure cineole were calculated from release data using Higuchi's equation (Table 1). Results revealed no significant differences (P ≤ 0.05) among cineole-treated matrices regarding diffusion coefficient. The diffusion coefficient of diazepam in pure cineole seems slightly higher, but the differences were insignificant (P ≤ 0.05).

**Table 1.** Diffusion Coefficients of Diazepam from Model Matrices, Containing Different Amounts of Cineole and Pure Cineole (n = 3) ^a^

Cineole Content (%w/w)	Diffusion Coefficient (cm^2^h^-1^) × 10^6^
**40**	5.11 ± 0.74
**60**	4.15 ± 1.09
**90**	4.25 ± 0.21
**100**	7.61 ± 2.71

^a^ Data are presented as mean ± SD.

### 4.6. Skin Permeation Studies

Results showed that the permeability coefficient of diazepam from matrices through rat skin increased significantly (P ≤ 0.05) by increasing cineole concentration in matrices from 40 to 60%, 90%, and then to pure cineole (Table 2). Results also revealed that both diffusion coefficient (estimated indirectly from lag-time) and partition coefficient play roles in rises in permeability coefficients.

**Table 2.** Permeability Coefficient (Kp) and Other Permeation Parameters of Diazepam from Cineole-treated Matrices and Pure Cineole Through Rat Skin at 32°C (n = 3) ^a^

Cineole Concentration (%w/w)	Kp (cmh^-1^) × 10^4^	Lag-time (h)	Kp Ratio	Lag-time Ratio	Difusivity Ratio*	Partitioning Ratio**
**40**	1.75 ± 0.29	4.36 ± 0.60	1.00	1.00	1.00	1.00
**60**	1.68 ± 0.26	4.00 ± 0.58	0.96	0.92	1.09	0.88
**90 **	3.31 ± 0.39	3.17 ± 0.37	1.89	0.73	1.37	1.38
**100**	7.27 ± 1.33	3.13 ± 0.31	4.15	0.72	1.39	2.98

^a^ Data are presented as mean ± SD. * Reciprocal of lag-time ratio. ** Calculated from Kp ratio and diffusivity ratio.

## 5. Discussion

### 5.1. X-ray Diffraction Studies

Small-angle reflections (2θ < 10°) provide information about the symmetry and dimension of the lattice, while reflections in the high-angle regions provide information about the conformation of hydrocarbon chains (3, 21, 27, 28). Reflections at 41.45 and 21.23 Å and also at 32.29, 16.62, and 8.69 Å of the matrix have a ratio of 1:1/2 (Figure 2), which is characteristic of lamellar structure (27, 29). The reflection of 4.18 Å may indicate the hexagonal alkyl chain packing in the model matrix (21, 23, 30). Although emerging a diffuse band at high angles indicates a disordered structure of hydrocarbon chains, the appearance of sharp reflections in this region at 2θ of 21.2°, 22.8°, and 24.2° (Figure 2) points that some of the hydrocarbon chains are in a stiff condition (21, 23). These results indicate a combination of gel-liquid crystalline lamellar arrangement in the matrix, in good agreement with Moghimi et al. (21).

Adding cineole (40, 50, and 60%) reduced the intensity of sharp reflections in the small-angle region, characteristic of ordered structure, while the diffuse band, characteristic of amorphous structure, became more prominent (Figure 2). In matrices containing higher amounts of cineole (70, 80, and 90%), the intensity of sharp reflections reduced dramatically, and the diffuse band in the high-angle region became greater (Figure 2), indicating losing the arrangement of plates and more fluidization of the system.

### 5.2. Polarized Light Microscopy

The model matrix showed oily streaks and mosaic textures at room temperature (Figure 3A), characteristic of lamellar structure arrangements (21). In matrices containing 25% cineole, mosaic texture, and positive units started growing and replaced the oily streaks (Figure 3B). These changes increased in matrices containing 40, 50, 60, and 70% cineole (Figure 3C, D, E, and F), indicating a more fluidized model matrix, in agreement with Moghimi et al. (21). At these cineole concentrations, some myelinic figures gradually appeared and developed (Figure 3C), characteristics of lamellar structure arrangements (21, 22). Myelinic figures, reported by Moghimi et al. (22) at 40% cineole, are tubular structures reported for the first time by Virchow (as cited in Boullerne) (31). These structures are stacked bilayers of amphiphiles, alternating with water layers, concentrically wrapped around a rod-like core axis (32, 33). Furthermore, at these cineole concentrations, spherulites, which are concentric lamellar structures, are also observed (Figure 3C, D, and E). These spherulites were reported by Moghimi et al. (21) for a 40% cineole-containing matrix, as well.

By increasing cineole to 80% and 90%, the matrix became two-phasic, a liquid phase, and a bulky mass. In the liquid phase, many positive units were observed (Figure 3G and J). These results agree with X-ray results, in which the system lost its order by increasing the amounts of cineole and became more amorphous. Oily streaks textures in the bulky mass of matrices containing 80% and 90% cineole (Figure 3H and I) indicate that the matrix has a stacked structure of lamellar arrangements in this region.

### 5.3. Differential Scanning Calorimetry

The first endothermic transition (around -10°C) that is seen as a shoulder of the second transition temperature (Figure 4) is probably due to the melting of linoleic acid (melting point -12°C) (21). The second transition found near 0°C (Figure 4) should be due to the melting of ice (frozen free water) in the matrix structure. The endothermic transition at around 40°C (Figure 4) is probably the consequence of structural changes in the lipid matrix, as described by Moghimi et al. (21) in a way that some oily streaks and planar areas become positive units and mosaic texture. Three other transitions at around 50, 80, and 110°C with very low enthalpies were also reported by Moghimi et al. (21), which are not visible in the thermogram of Figure 4, apparently due to very low intensities.

Adding cineole to the model matrix caused a gradual diminishing in the intensity of the main structural transition temperature at around 40°C (Figure 4, insert box), in good agreement with polarized light microscopy and X-ray studies that indicate matrix fluidization upon cineole addition. These results are also in agreement with our previous work (22), which showed that increasing the cineole content of the model matrix to 25% and 40% causes severe fluidization and corresponding changes in their DSC thermogram, while the transition of around 40°C vanishes at 40% cineole content.

### 5.4. Release Studies

Release experiments showed that diffusion coefficients remained almost constant even by increasing the cineole content of the model matrix to 60% and 90% (P ≤ 0.05) (Table 1). This result suggests that matrices at 40% cineole and more are almost identical in fluidization. In our previous work (17, 18), diffusion coefficients of hydrophilic and lipophilic drugs increased by increasing cineole concentration in a concentration-dependent manner in matrices containing 5 - 25% cineole, where the matrix was not fully fluidized yet. The present results suggest an optimum concentration of cineole for full fluidization of the matrix. However, we should note that drug entrapment inside fine structures of fluidized matrices might have counterbalanced fluidization by changing the effective drug concentration.

### 5.5. Skin Permeation Studies

Skin permeation results (Table 2) showed that by increasing the cineole content of the matrix, the permeability coefficient (Kp) increased, lag-time decreased, and, therefore, diffusion coefficients increased. Data also showed that Kp alterations were due to both diffusion coefficient and partition coefficient changes in a concentration-dependent manner (Table 2). In agreement with the present results, we previously showed that the presence of cineole in the matrix increases the diffusivity of the model lipophilic drug (estradiol) in the model matrices containing 5 - 25% cineole (17) and that the increased permeation is due to both diffusion coefficient and partition coefficient increments (18). However, as discussed above for the present release studies, the diffusion coefficient of diazepam in matrices containing 40, 60, and 90% cineole remained constant (Table 1). Therefore, the present skin data showed that high concentrations of cineole at the surface (in matrices or as pure cineole) did not provide enough cineole in the skin for the full fluidization of intercellular lipids. This is in agreement with our previous calculations (22) using Cornwell et al. data (10), indicating that the cineole content of the intercellular lipids of the SC after treatment of the whole SC with pure cineole might not reach such high values of 50% or higher. It is also possible that mechanical or geometrical hindrances and attachment of intercellular lipids to coenocytes' proteins (lipid-protein envelope) prevent the full fluidization of intercellular lipids. All of these require further investigations.

### 5.6. Conclusions

The results of the present model matrix revealed that increasing cineole content increased the fluidity and lipophilicity of stratum corneum lipids, and new structures in the intercellular lamellar lipids were produced. Release data showed that fluidization reached almost its maximum at around 40% cineole content, while the permeation data showed that, apparently, it did not. In other words, the present skin data showed that high concentrations of cineole at the skin surface did not necessarily provide enough cineole concentration in the skin for the full fluidization of intercellular lipids, which is very important for adjusting enhancer concentration in vehicles during drug delivery system design. Other mechanisms, such as geometrical or structural hindrances, might also prevent the full fluidization of intercellular lipids at intermediate concentrations, all of which require further investigation.

## Data Availability

The data presented in this study are uploaded during submission as part of the manuscript as tables and figures.
